# The Dual Regulation Effects of ESR1/NEDD4L on SLC7A11 in Breast Cancer Under Ionizing Radiation

**DOI:** 10.3389/fcell.2021.772380

**Published:** 2022-02-16

**Authors:** Rui Liu, Lin Liu, Yan Bian, Shinan Zhang, Yue Wang, Huajian Chen, Xinyue Jiang, Guanghui Li, Qing Chen, Chang Xue, Mengke Li, Lianchang Liu, Xiaodong Liu, Shumei Ma

**Affiliations:** ^1^ School of Public Health and Management, Wenzhou Medical University, Wenzhou, China; ^2^ NHC Key Laboratory of Radiobiology, School of Public Health of Jilin University, Changchun, China; ^3^ The Second Affiliated Hospital of Jilin University, Changchun, China; ^4^ Key Laboratory of Watershed Science and Health of Zhejiang Province, Wenzhou Medical University, Wenzhou, China

**Keywords:** ESR1, SLC7A11, ionizing radiation, NEDD4L, ferroptosis, breast cancer

## Abstract

Radiotherapy is one of the most important treatments for breast cancer. Ferroptosis is a recently recognized form of regulated cell death that is characterized by lipid peroxidation. However, whether ionizing radiation (IR) could induce ferroptosis in breast cancer and how it works remain unknown. Bioinformatics analysis were performed to screen ferroptosis-related genes differentially expressed in breast tumor tissue and normal tissue. Then, breast cancer cell lines with different estrogen receptor (ER) phenotypes were used for studies *in vitro*, including ER-positive (MCF-7 and ZR-75-1) and ER-negative (MDA-MB-231) cells. The dynamic changes of mRNA and protein levels were examined after x-ray of 8 Gy by qRT-PCR and Western blotting, respectively. Immunoprecipitation (IP) was used to explore the interaction between proteins. Luciferase assay was used to analyze the transcriptional regulation effect of ESR1 on SLC7A11. BODIPY C11 and trypan blue dyes were used to determine lipid peroxidation and cell death, respectively. The result showed that the ferroptosis-related gene SLC7A11 was higher in breast cancer tissues compared with normal tissues and associated with poor survival. A positive correlation exists between ESR1 and SLC7A11 expression. ESR1 promoted SLC7A11 expression at the early stage after IR. ESR1/SLC7A11 knockdown significantly enhanced IR-induced ferroptosis in ER-positive cells. At 12 h after IR, the IP data showed the interaction between E3 ubiquitin ligase NEDD4L and SLC7A11 increased, followed by the ubiquitylation and degradation of SLC7A11. Thus, SLC7A11 expression was regulated by both ESR1 and NEDD4L, in opposite ways. For the first time, we elucidated that ESR1 and NEDD4L functioned together after radiation treatment and finally induced ferroptosis in breast cancer cells, which provides novel insight into the guidance of clinical treatment of breast cancer.

## Introduction

Breast cancer is a complex and clinically heterogeneous disease. Similar clinicopathologic characteristics and histologic types may have different clinical course and survival rates, and distinct molecular subtypes of tumors are inextricably bound up with clinical outcomes (Kumar Atri et al., 2017; Lian et al., 2017). According to the expression status of progesterone receptor (PR), estrogen receptor (ER), and human epidermal growth factor receptor 2 (HER2), immunohistochemistry is often used to characterize four subtypes: luminal A (ER^+^ and HER2^−^), luminal B (HR^+^ and HER2^+^), triple-negative/basal-like (ER^−^, PR^−^, and HER2^−^), and HER2-enriched (ER^−^, HER2^+^, and PR^−^) (Meyers et al., 2011). ERα and Erβ are two main forms of ER, encoded by separate genes (ESR1 and ESR2), respectively. Clinically, ESR1 is used to define the ER status of breast cancer (Lin et al., 2016). It has been reported that 70% of breast cancer are ER-positive at diagnosis (Khan et al., 2019). Under these circumstances, high expression of ER functions in tumor development and progression (Stoner et al., 2002; Ferreira Almeida et al., 2020). ER is a ligand-activated transcription factor involved in the modulation of cell division, which is vital to cell growth and survival (Cho et al., 2005; Carroll, 2016).

Ferroptosis is a novel type of programmed cell death distinct from apoptosis, necrosis, and autophagy, which is an iron-dependent form of regulated cell death involving accumulation of reactive oxygen species (ROS) and overwhelming lipid peroxidation (Stockwell et al., 2017). SLC7A11 is one of the critical regulators of ferroptosis, which promotes cystine uptake and glutathione biosynthetic and is used to generate the anti-oxidant glutathione (GSH), thus converting lipid hydroperoxides to lipid alcohol to protect cells from death (Liu et al., 2021). Furthermore, IR induces ferroptosis in cancer patients and improves the therapeutic effects and survival of radiation therapy (Wu et al., 2020a; Lei et al., 2020). Recently, studies have revealed that IR induces ferroptosis by the suppression of SLC7A11, which represents an important part of radiotherapy (Lang et al., 2019). It is also shown that IR induces the expression of SLC7A11, as an adaptive response (Lei et al., 2020). Many studies have indicated that ferroptosis also mediated cell death in breast cancer by SLC7A11 (Xu et al., 2020; Yang et al., 2021), which implied the important role of SLC7A11 in regulating the ferroptosis of breast cancer. However, the mechanisms for IR-induced ferroptosis and regulation in breast cancer cells remain largely unknown.

Ionizing radiation (IR) can directly interact with water in human bodies, and generate ROS, which could be amplified by Fenton reaction and generate an extremely reactive hydroxy radical (Ahsan et al., 2003; Yu, 2012). The hydroxy radical results in lipid peroxide and induces cell death, including ferroptosis (Kajarabille and Latunde-Dada, 2019). Radiotherapy is an essential mode for many cancers, including breast cancer, particularly those seeking breast-conserving surgery. Radiotherapy to breast conservation surgery can halve the rate of disease recurrence and reduce breast cancer mortality by about one-sixth (Early Breast Cancer Trialists’ Collaborative Group (EBCTCG) et al., 2011). According to NCCN clinical practice guidelines in oncology, the adjuvant therapy and overall treatment pathway should be adjusted according to the level of ER expression, although in the ER-positive group (Gradishar et al., 2020). In this study, we designed a systematic study to elucidate the underlying mechanisms of radio-resistance in ER-positive breast cells and managed to improve radiation killing effect through targeted intervention of IR-induced ferroptosis.

## Materials and Methods

### Data Collection

The gene expression profiles ( raw counts and FPKM-UQ) were downloaded from the TCGA database (https://gdc-portal.nci.nih.gov) by the TCGA biolinks R package (version 2.20.0) (Colaprico et al., 2016). Data from metastatic, male, or formalin-fixed paraffin-embedded tissue samples, as well as repeated data for the same samples, were excluded. After exclusion, 1,191 primary solid tumor (TP) and 112 solid tissue normal (NT) samples were retained for genomic data. The survival and clinical information of breast cancer were obtained from the Xena Functional Genomics Explorer of University of California Santa Cruz (https://xenabrowser.net/).

### Reagents, Antibodies, and Plasmids

Monoclonal anti-FLAG antibody and anti-β-actin were obtained from Sigma (St. Louis, United States). Rabbit polyclonal anti-SLC7A11, and rabbit polyclonal anti-ESR1 were from Cell Signaling Technology (CST, United States). Protein G Plus-agarose was from Sangon Biotech (Shanghai, China). RNAiso Plus kits, reverse transcription kits, and real-time PCR kits were from Takara Biomedical Technology (Dalian, China). Ferrostatin-1 (Fer-1), Erastin, and carbobenzoxy-Leu-Leu-leucinal (MG132) were all purchased from MedChemExpress (Shanghai, China). The expression plasmids for MYC-Ub were from Dr. Haishan Huang’s lab (Wenzhou Medical University, China). SLC7A11 and ESR1 genes were obtained by PCR using MCF-7 cDNA as a template and then inserted into pcDNA3.1-FLAG vectors.

### Cell Culture and siRNA, Plasmid Transfection

The human-source cell lines MCF-7, ZR-75-1, and MDA-MB-231 were obtained from the Cell Bank of Chinese Academy of Sciences and periodically tested for the absence of *mycoplasma*. The cell lines were grown in high-glucose DMEM medium (Gibco, United States) supplemented with 10% fetal bovine serum and 1% penicillin and streptomycin in a 37 C incubator maintained at 5% CO_2_. For transfection, when the cells had grown to 70%–80% confluence in six-well plates, the plasmid (1 μg) or siRNA (100 μM) was transfected using Lipofectamine 2000 (Invitrogen, United States) according to the manufacturer's instructions. The siRNAs were synthesized by GenePharma (Shanghai, China), and the sequences were listed as follows: siRNA for ESR1, CTA​CAG​GCC​AAA​TTC​AGA​TAA; NEDD4L, CGC​CTT​GAC​TTA​CCT​CCA​TAT.

### Flow Cytometric Analysis

Cell death and lipid peroxidation were assessed by flow cytometry with trypan blue (Gibco, United States) and BODIPY™ 581/591 C11 (Thermo Fisher Scientific, United States) following the manufacturer’s instruction, respectively. In brief, cells were pretreated with 10 µM of Erastin or 10 µM of Fer-1 for 1 h and then exposed to IR. Cells were collected at 48 h for lipid peroxidation or 72 h for cell death testing after X-ray exposure. Cells were resuspended in 400 μl of serum-free medium that contained BODIPY™ 581/591 C11 (5 µM) at 37°C in the dark for 30 min, or in 200 μl of PBS containing 0.04% of trypan blue, respectively to detect lipid peroxidation and cell death. The data were analyzed using the NovoExpress software (ACEA, United States), and a minimum of 10,000 cells were analyzed per condition.

### Western Blot and Immunoprecipitation

For Western blot, cells were lysed in ice-cold RIPA Lysis Buffer. Lysates were centrifuged at 12,000 rpm for 15 min at 4°C. The supernatant was collected, and the protein content was determined using the BCA Protein Assay Kit (Thermo-Fisher, United States) according to the manufacturer’s instructions. Equal amounts of total protein (20 μg) were separated by 10% or 12% SDS–PAGE and then transferred to an equilibrated PVDF membrane (Millipore, United States). β-actin was used as a loading control. For IP, MCF-7 cells were transfected with expression vectors for FLAG-SLC7A11, and were exposed to IR at 48 h post-transfection. At 24 h after IR, cells were lysed in 1% NP-40 lysis buffer. Whole cell lysates (WCL) were incubated with the specified antibodies overnight at 4°C with constant rotation. Then, protein G Plus-agarose was added for 2 h under rotation at 4°C. After incubation, immunocomplexes were washed five times with IP wash buffer (Sangon Biotech, China) and subsequently resolved by SDS-PAGE followed by Western blot analysis.

### Ubiquitination Assay

Cells were transiently transfected with MYC-tagged ubiquitin (UB), and FLAG-tagged SLC7A11 with or without si-NEDD4L for 48 h, and were exposed to IR for 24 h. At 24 h after IR, cells were pretreated with MG132 (10 μM) for 4 h before harvest. The lysates were subjected to immunoprecipitation with FLAG antibodies overnight at 4°C. Ubiquitinated proteins were determined by immunoblotting with anti-MYC antibodies (Santa Cruz, United States).

### Real-Time PCR for mRNA Quantification

Total RNA was isolated from cultured cells using RNAiso Plus kits according to the manufacturer’s instructions. mRNAs were first reverse transcribed into cDNA and quantitative reverse transcription-PCR (qPCR) was performed as the following thermal conditions: 95°C for 30 s followed by 40 cycles of 95°C for 5 s and 60°C for 30 s. qPCR was performed on QuantStudio 3 (Applied Biosystems, United States), and the expression of target genes was normalized to that of GAPDH. The following primers were used: GAPDH forward CCA​TGG​GTG​GAA​TCA​TAT​TGG​A and reverse TCA​ACG​GAT​TTG​GTC​GTA​TTG​G; SLC7A11 forward GCG​TGG​GCA​TGT​CTC​TGA​C and reverse GCT​GGT​AAT​GGA​CCA​AAG​ACT​TC; and ESR1 forward GCT​TAC​TGA​CCA​ACC​TGG​CAG​A and reverse GGA​TCT​CTA​GCC​AGG​CAC​ATT​C.

### Double Fluorescein–Reporter Enzyme

Double luciferase–reporter gene determination was conducted to determine whether SLC7A11 the downstream target gene of ESR1. Reporter plasmid of SLC7A11 promoter double luciferase was synthesized from Generalbiol (Anhui, China) and inserted into the pGL3-Basic vector. pGL3-Basic SLC7A11 promoter was co-transfected into cells with ESR1 overexpression vector or control vector by Lipofectamine 2000. After transfection for 48 h, luciferase activity was determined on a Centro LB 960 Luminometer (Berthold Technologies, Germany) and the activity of renilla luciferase was used as a standardized control.

### Statistical Analysis

Statistical analysis was performed using R version 4.1.1 (http://www.r-project.org/) and GraphPrism 5.0 software (GraphPad Software, United States). The differentially expressed gene (DEG) analyses were done with R package edgeR (version 3.34.0) (Robinson et al., 2010). According to the suggested workflow, raw RNA-Seq read count data were normalized by Trimmed-mean *M* values (TMM) to balance the counts among the different samples (Maza, 2016). Genes were marked as DEGs if the absolute value of log_2_ transformed fold change (log_2_FC) ≥ 1.5 and the false discovery rate (FDR) < 10^-10^. The FPKM-UQ normalized gene expression data were log-transformed for further analyses. The R package survminer (version 0.4.9) was used to separate patients into higher and lower expression groups by gene expression. The relationship between SLC7A11 expression status and clinicopathologic characteristics of breast cancer was analyzed by Chi-square test. The association with prognosis of breast cancer patients was determined by Kaplan–Meier survival analysis with Log-rank test. A Cox proportional hazard regression model was conducted to perform a univariate and multivariate analysis. All experiments were performed in triplicate, and replicate data were used to calculate means ± standard deviation (SD). ANOVA plus subsequent Dunnett post hoc tests were performed for multiple comparisons. If not specified above, a *p*-value less than 0.05 was considered to be statistically significant, and *p*-values were two-tailed.

## Result

### Differential Expression of Ferroptosis-Related Genes in Breast Tumor Samples

**FIGURE 1:**
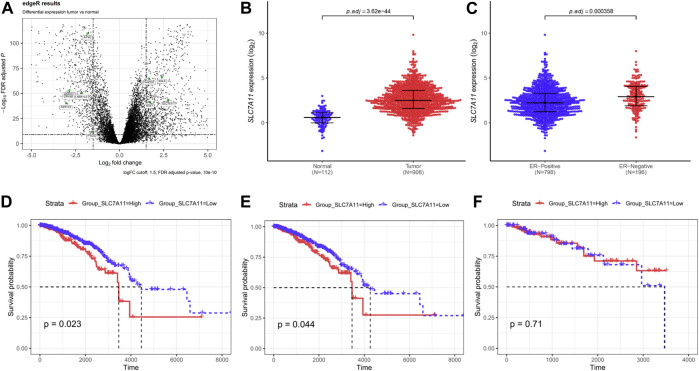
Compared with normal tissues, ferroptosis-related gene SLC7A11 was upregulated in breast cancer tissues and contributed to poor survival. **(A)** The differentially expressed genes (DEGs) were identified between TP and NT with in breast cancer patients. Volcano plot shows the gene statistical significance with |log2FC| > 1.5 and FDR <10^-10^. The green points were ferroptosis-related genes. **(B,C)** Comparison of SLC7A11 expression in different subgroups. Normal and tumor **(B)**; ER-positive and ER-negative **(C)**. (**D–F**) Kaplan–Meier curves of overall survival (OS) in breast cancer according to SLC7A11 expression. Overall survival analysis **(D)**; Subgroup analyses of OS in ER-positive **(E)** and ER-negative **(F)**.

Breast cancer data were collected from TCGA. Based on the screening principles, 1191 TP and 112 NT samples were selected into this study. DEGs were reflected in volcano plot (Figure 1A). According to the principle of statistics, 4 upregulated and 6 downregulated ferroptosis-related DEGs were associated with breast cancer. Among them, SLC7A11 was a critical regulator of ferroptosis (Koppula et al., 2020). The SLC7A11 mRNA expression was extremely upregulated in tumor tissues compared with normal tissues (Figure 1B). Excluding unavailable SLC7A11 expression value, 1,104 breast cancer samples including 217 high expressions (19.66%) and low 887 expressions (80.34%) were extracted for further study. Chi-Square analysis were applied to study the data and results revealed that there were many clinicopathologic features significantly correlated with SLC7A11 expression, including age, histological type, molecular subtype, ER, PR, and N classification (Supplementary Table S1). Differences in SLC7A11 expression are shown in Figure 1C according to ER subtype. SLC7A11 expression was lower in ER-positive tissues than ER-negative tissues.

### The High Expression of SLC7A11 Predicted Poor Prognosis in Breast Cancer

In univariate Cox regression for overall survival (OS) model, we included variables that correlated with SLC7A11. The following multivariate analyses confirmed SLC7A11 expression, age, and N classification as independent prognostic factors after adjusting other prognostic indicators (Supplementary Table S2). Analysis demonstrated that patients with higher SLC7A11 were associated with worse OS than those with lower SLC7A11 (Figure 1D). In addition, K–M curves were used to evaluate the prognostic value in differentiation ER subtypes, which revealed that SLC7A11 specifically associated with OS in ER-positive cancers (Figure 3E), but not in ER-negative cancers (Figure 1F).

Then, we further define the relationship between ferroptosis and ER-positive cancers, and eight ferroptosis-related genes were analyzed according to Wu et al. (2020b) and Tang et al. (2021). Results revealed that only low expression of SLC7A11 supported ferroptosis occurrence in ER-positive cancers (Supplementary Figure S1). The above data suggest that SLC7A11 may play an important role in the regulation of ER-positive cell ferroptosis.

### IR Induced Ferroptosis by the Reduction of SLC7A 11 Expression

**FIGURE 2:**
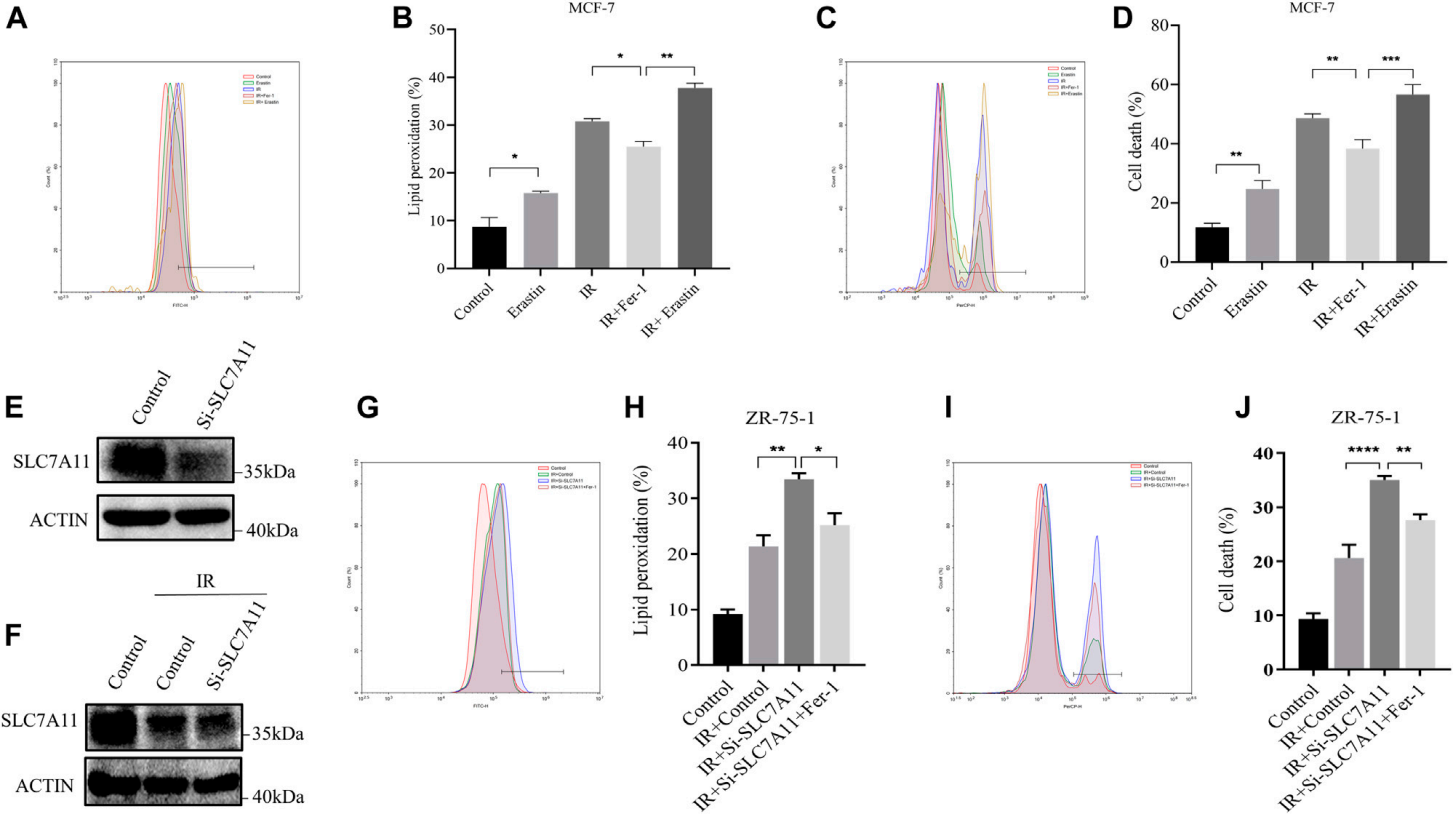
IR-induced ferroptosis in ER-positive breast cell lines. **(A–D)** MCF-7 cells were pretreated with Erastin (10 µM) or Fer-1 (10 µM) for 1 h and then exposed to IR (8 Gy). **(E–J)** Western blot and flow cytometry analysis of ferroptosis by SLC7A11 downregulation in ZR-75-1. Lipid peroxidation **(A,B,G,H)** and cell death **(C,D,I,J)** were assessed by flow cytometry using C11-BODIPY and trypan blue, respectively. Shown is mean ± SD, *n* ≥ 3, *p < 0.05; **p < 0.01; ***p < 0.001; ****p < 0.0001.

The IR-induced ferroptosis in different breast cancer cell lines were evaluated in our previous study (Ma et al., 2021). Fer-1 was able to significantly attenuate IR-induced cell death in ER-positive MCF-7 and ZR-75-1, but not in ER-negative MDA-MB-231 cells. To further verify the result, we analyzed the effects of Erastin and Fer-1 on lipid peroxidation accumulation and cell death in response to IR treatment. Importantly, Fer-1 significantly reduced IR-stimulated lipid peroxidation and cell death. Correspondingly, Erastin significantly increased lipid peroxidation accumulation and cell death, and showed synergistic effect with IR (Figures 2A–D). When we knockdown of SLC7A11 in ZR-75-1, IR-induced lipid peroxidation and cell death significantly increased, which could be rescued by Fer-1 (Figures 2E–J). These results suggested that ferroptosis was one pattern of IR-induced cell death in ER-positive cells, and SLC7A11 may play an important role in this process.

### ESR1 Activates the Transcription of SLC7A11

**FIGURE 3:**
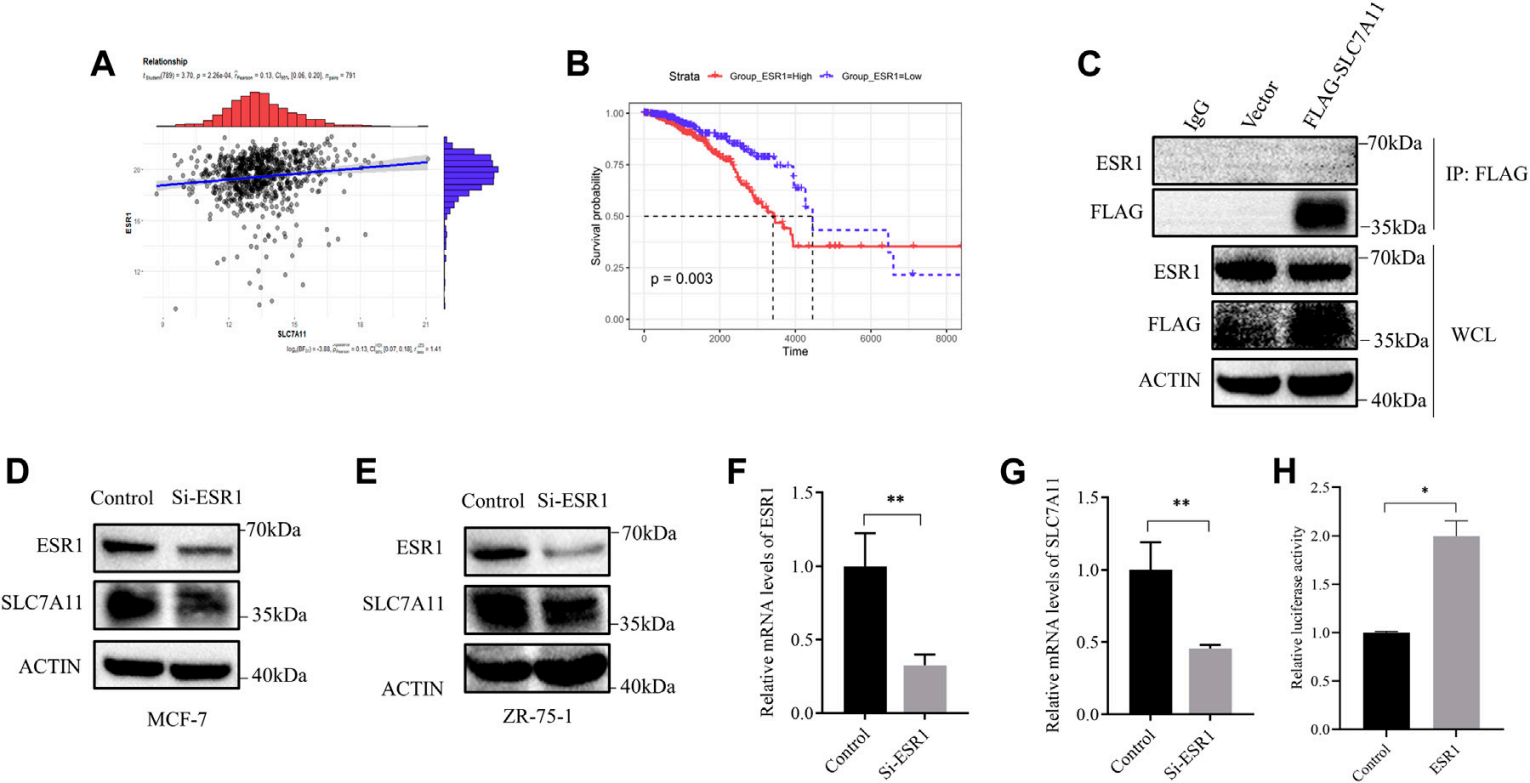
Correlation between ESR1 and SLC7A11 in ER-positive cancers. **(A)** Scatter plots for correlation analysis of ESR1 and SLC7A11. The dark gray line means a linear regression line and the gray region is 95% confidence region. **(B)** Kaplan–Meier curve of OS in ER-positive cancers according to ESR1 expression. **(C)** IP assay of MCF-7 cells transfected with a plasmid expressing FLAG-tagged SLC7A11. **(D–G)** Western blot and qRT-PCR analysis showed that si-ESR1 reduced SLC7A11 expression in MCF-7 **(D,F,G)** and ZR-75-1 **(E)**. **(H)** Effect of ESR1 on SLC7A11 transcription by double fluorescein-reporter enzyme activity in MCF-7. Shown is mean ± SD, *n* ≥ 3, *p < 0.05; **p < 0.01.

Referring to the above results, we considered that the relationship between SLC7A11 expression and breast cancer survival was probably based on ESR1. Furthermore, a positive correlation of ESR1 with SLC7A11 was found in ER-positive cancers (Figure 3A) and the survival curve of ESR1 expression in ER-positive cancers is shown in Figure 3B. From the K–M curve, higher expression of ESR1 was also related to poor prognosis. From these results, we can conclude that SLC7A11 and ESR1 are both related to survival in ER-positive cancers. However, the precise relationship is unclear.

Then, we designed siRNA targeting ESR1 to verify the function of ESR1. To determine whether ESR1 could interact with SLC7A11, an IP experiment was carried out. Our result suggested that there was no direct protein–protein interaction (Figure 3C). In order to determine the relationship between ESR1 and SLC7A11, MCF-7 and ZR-75-1 cells were transfected with siRNA-NC or siRNA-ESR1; 72 h later, cells were harvested, and the total mRNA and proteins were extracted and subjected to qPCR and Western blotting, respectively. As shown in Figures 3D–G, si-ESR1 significantly reduced ESR1 mRNA and protein levels, following the SLC7A11 levels decreased. Further dual-luciferase reporter gene assay was applied and the results showed that EGR1 was capable of activating the SLC7A11 promoter ESR1 was able to activate the transcription of SLC7A11 (Figure 3H). These findings support that ESR1 suppresses ferroptosis by promoting SLC7A11 expression in IR treatment.

**FIGURE 4:**
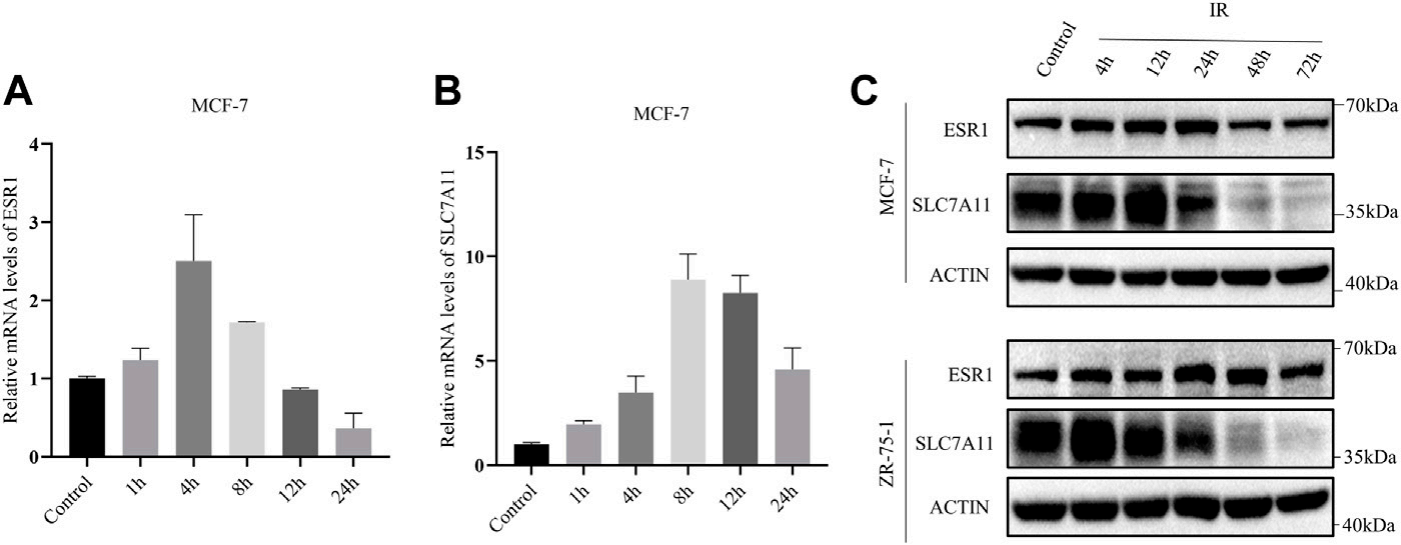
IR-induced SLC7A11 and ESR1 expression in ER-positive cell lines. Cells were collected at different time points after IR with 8 Gy x-rays. Western blot, and qRT-PCR were used to detect the expression of SLC7A11 and ESR1. **(A,B)** Relative mRNA expression of ESR1 **(A)** and SLC7A11 **(B)** were detected in MCF-7. Shown is mean ± SD. **(C)** SLC7A11 and ESR1 protein expression by IR treated in MCF-7 and ZR-75-1.

In addition, the mRNA levels of SLC7A11 and ESR1 were evaluated by qRT-PCR based on time course. After exposure to X-ray, there was an increase in the mRNA levels of SLC7A11 and ESR1 (Figures 4A,B). The protein levels of SLC7A11 and ESR1 based on IR time course were evaluated by western blot (Figure 4C). The results suggested that IR-induced ferroptosis occurred at 48 h, but at 4–12 h SLC7A11 had a rising period to against cell death.

### ESR1 Functions as a Cell Survival Response in Early Effects of IR

**FIGURE 5:**
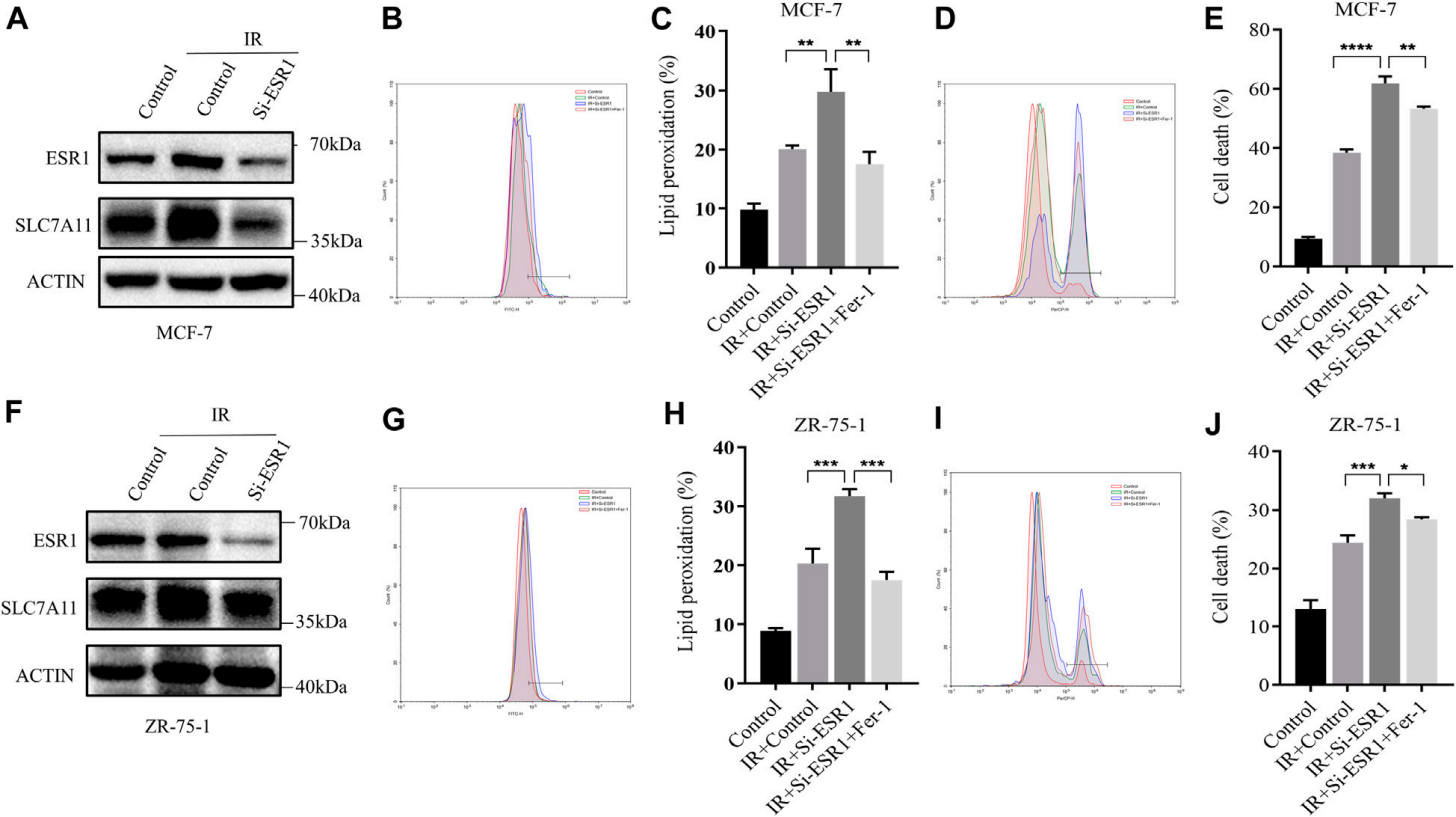
ESR1-SLC7A11 axis is involved in IR-induced ferroptosis. The results of Western blot assay show that ESR1 knockdown inhibited the increase of SLC7A11 induced by IR at 12 h in MCF-7 **(A)** and ZR-75-1**(F)**. **(B–E,G–J)** Lipid peroxidation and cell death were assessed by flow cytometry using C11-BODIPY and trypan blue, respectively. Shown is mean ± SD, *n* ≥ 3, *p < 0.05; **p < 0.01; ***p < 0.001; ****p < 0.0001.

Lipid peroxidation and cell death were assayed by flow cytometry using the fluorescent probes C11-BODIPY and trypan blue, respectively. ESR1 knockdown rendered ER-positive cells susceptible for ferroptosis and lipid peroxidation accumulation induced by IR. When ESR1 was knockdown, the increase of SLC7A11 induced by IR was shown to significantly attenuate in MCF7 (Figure 5A) and ZR-75-1 (Figure 5F). The suppression of ESR1 by siRNA increased IR-induced cell death as well as rise of lipid peroxidation in MCF-7 (Figures 5B–E). Moreover, IR had similar effects on cell death and lipid peroxidation with ESR1 knockdown in ZR-75-1 (Figures 5G–J). Indeed, ESR1 knockdown showed a significant effect on ferroptosis induced by IR. The increased lipid peroxidation and cell death can be significantly rescued by Fer-1.

**FIGURE 6:**
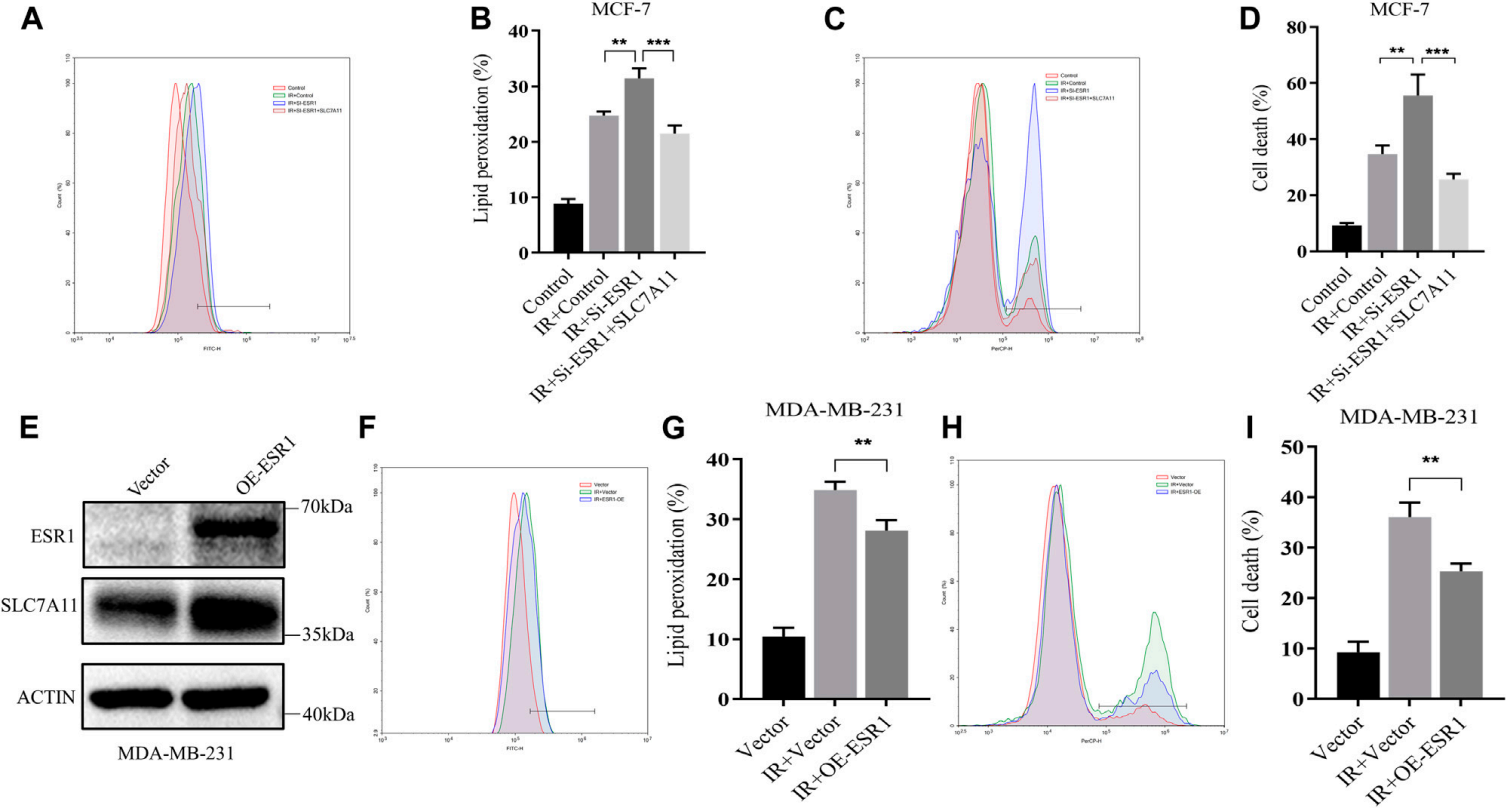
Characterization of rescue cells and ESR1 overexpression. **(A–D)** MCF-7 cells were co-transfected with Si-ESR1 and pcDNA3.1-SLC7A11 for 48 h, then treated with IR (8 Gy). **(E)** Western blotting analysis of ESR1 overexpression in MDA-MB-231. **(A–D, F–I)** Lipid peroxidation and cell death were assessed by flow cytometry using C11-BODIPY and trypan blue, respectively. Shown is mean ± SD, n ≥ 3, **p < 0.01; ***p < 0.001.

On the basis of these findings, a rescue assay was conducted to confirm the role of ESR1 in SLC7A11-mediated IR-induced ferroptosis. As shown in Figures 6A–D, SLC7A11 can also rescue siESR1-increased lipid peroxidation and cell death. Over-expression of SLC7A11 rescue IR-induced ferroptosis in ESR1 knockdown cells.

For further verification of the ESR1 effect, we overexpressed ESR1 in ER-negative cells. The data showed that the upregulation of ESR1 could increase SLC7A11 (Figure 6E) and rescue IR-induced lipid peroxidation (Figures 6F,G) and cell death (Figures 6H,I). Our results indicate that ESR1 promotes SLC7A11 transcription to resistance ferroptosis and cytotoxic stress, thereby promoting tumor malignancy.

### The NEDD4L-SLC7A11 Interaction Increased After Radiation Treatment

**FIGURE 7:**
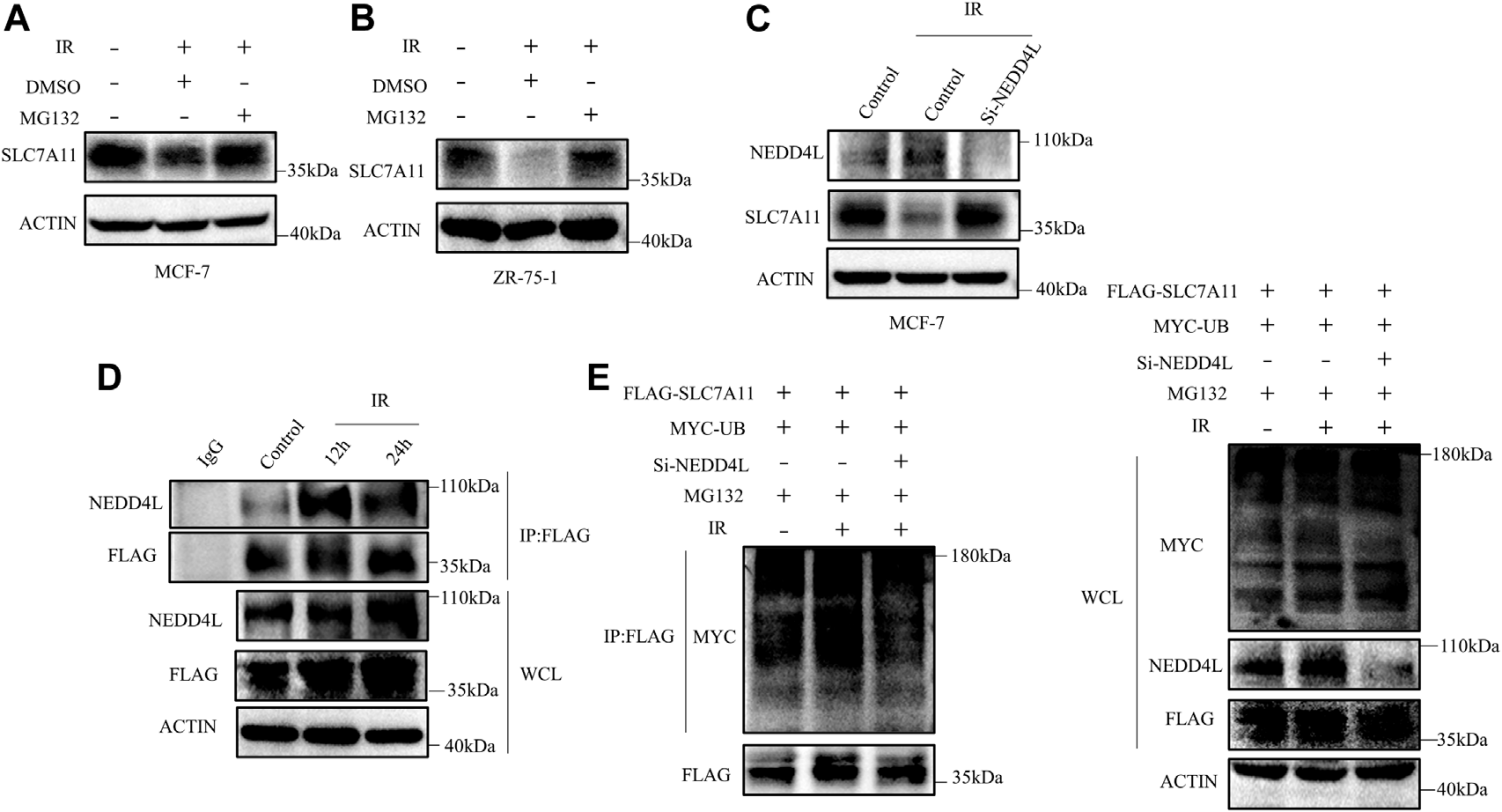
SLC7A11 is degraded by ubiquitination after IR treatment. MCF-7 **(A)** and ZR-75-1 **(B)** cells were pretreated with MG132 (10 μM) or DMSO for 4 h before harvest. **(C)** MCF-7 cells were transfected with Si-NEDD4L. The protein level of SLC7A11 was analyzed by Western blot. **(D)** Western blot analysis of WCL and IP samples of IgG or anti-NEDD4L antibody were conducted to detect the interaction between SLC7A11 and NEDD4L. MCF-7 cells were transfected with FLAG-SLC7A11 for 48 h, then treated with IR. MG132 (10 μM) was added into the culture medium for 4 h before harvest. **(E)** Western blotting analysis of WCL and ubiquitin IP samples were performed with indicated antibody in MCF-7 cells. The cells were treated with MG132 (10 μM) for 4 h before harvest.

We next detected whether the reduction of SLC7A11 was regulated at the transcriptional level or posttranscriptional level. The mRNA and protein levels of SLC7A11 inconsistently changed in IR treated at 24 h (Figure 4B), indicating that IR regulated the expression of SLC7A11 post-transcriptionally. To test this assumption, MG132, which acts as a blocker in the ubiquitin–proteasome pathway, was used and we found that SLC7A11 expression was accumulated by MG132 (Figures 7A,B). These observations suggest that SLC7A11 is regulated by the ubiquitin–proteasome pathway. By using the UbiBrowser database, NEDD4L is the one with the highest confidence to interact with SLC7A11 (Supplementary Table S3) and serves as a potential E3 ligase (http://ubibrowser.ncpsb.org.cn/ubibrowser/) (Li et al., 2017). Our data showed that NEDD4L knockdown rescued IR-induced degradation of SLC7A11 (Figure 7C). We then confirmed the interaction between SLC7A11 and NEDD4L by IP experiments, and a stronger binding was detected after IR treatment (Figure 7D), suggesting that NEDD4L associates with SLC7A11 in ER-positive cells. Consistent with these observations, we found that knockdown of NEDD4L markedly reduced the ubiquitination of SLC7A11 in ER-positive cells (Figure 7E). Taken together, IR could increase the interaction between NEDD4L and SLC7A11.

## Discussion

Ferroptosis is a form of programmed cell death driven by cellular metabolism and iron-dependent lipid peroxidation, but its role in breast cancer remains unclear. Our study found that SLC7A11 was correlative to survival in breast cancer and high expression implied poor prognosis. Moreover, in multivariate analysis, high expression of SLC7A11 was an independent prognostic factor for OS of breast cancer. The prognostic analysis of SLC7A11 in subtypes showed that SLC7A11 is specifically associated with OS in ER-positive cancers. It is reported that SLC7A11 plays an important role in the protection of cells from oxidative stress and ferroptosis by promoting the uptake of cystine and the synthesis of GSH (Stockwell et al., 2017; Koppula et al., 2020; Sun et al., 2021). Our research suggested that ferroptosis occupies a very important position in IR-induced cell death in ER-positive cell lines. We also found that ESR1 expression was also related to prognosis in ER-positive cancers, and our finding was consistent with Ogłuszka’s group, which reported that lowered expression of ESR1 was more favorable for disease-free survival (Ogłuszka et al., 2019). ESR1 is a member of the nuclear receptor superfamily; after the activation by estrogen, it can translocate into the nucleus and function as a transcription factor (Nilsson and Gustafsson, 2011; Lai et al., 2015). Among total breast cancer cases, 70% of cases are ER-positive (Khan et al., 2019). In addition, the presence of elevated levels of ESR1 in benign breast epithelium appears to indicate an increased risk of breast cancer, and the downregulation of ESR1 results in induction of autophagy and apoptosis (Ali and Coombes, 2000; Zheng et al., 2015). A recent report showed the association of ESR1 with ferroptosis in breast cancer, especially in cells with low ESR1 expression (Yu et al., 2019). The unclear relationship between ESR1 and SLC7A11 is puzzling and in great need of clarification.

Ferroptosis is characterized by the overwhelming iron-dependent accumulation of lipid hydroperoxides. Many reports provide definitive evidence for IR-induced lipid peroxidation, and ferroptosis is as important as other forms of cell death (Lang et al., 2019; Lei et al., 2020; Ye et al., 2020). IR induces or reduces the expression of ferroptosis inhibitor SLC7A11 to regulate ferroptotic cell death in different cell lines (Lang et al., 2019; Lei et al., 2020). Our present study finds that SLC7A11 correlated to IR-induced ferroptosis, with SLC7A11 transiently increased as an adaptive response and reduced continuously thereafter in ER-positive cell lines. It is important to emphasize that the mechanism of IR-induced ferroptosis is complicated. In this study, we focused on IR-induced ferroptosis regulated by SLC7A11. ESR1 is an estrogen-activated transcription factor and is responsible for transcription of cell-critical genes such as those involved in regulating proliferation (Khorshid Shamshiri et al., 2020). We detected SLC7A11 changes following ESR1 knockdown and found a positive correlation between ESR1 and SLC7A11 expression. Dual-fluorescence reporter assay confirmed that ESR1 upregulated SLC7A11 by promoting SLC7A11 gene transcription. ESR1 knockdown suppressed the IR-induced SLC7A11 transient increase. In addition, ESR1 knockdown also had a significant positive effect on ferroptosis. Based on the above results, ESR1 a central role in upregulating anti-ferroptosis defense SLC7A11 for ER-positive cancer in IR treatment. SLC7A11 was transiently increased after IR and reduced continuously thereafter. IR was able to increase the expression of ESR1, which activated SLC7A11 expression. ESR1 played a significant role in the initial stage against IR-induced ferroptosis. However, IR increased SLC7A11 protein degradation stronger than the protein synthesis in the long term, and then ferroptosis occurred. However, the exact regulatory mechanism underlying the SLC7A11-mediated ferroptosis after IR in breast cancer remains unknown and needs further study.

In this study, we further investigated the mechanism of SLC7A11 degradation induced by IR. NEDD4L is an E3 ubiquitin ligase, containing four WW domains for substrate interaction (Yang and Kumar, 2010). To the best of our knowledge, we report for the first time that NEDD4L binds to SLC7A11 for SLC7A11 degradation in IR treatment. A recent evidence suggests that NEDD4L likely displays maximal activities when localized to membrane (Escobedo et al., 2014). NEDD4L activity is influenced by the conformational changes, with specific interaction between phosphatidylinositol 4,5-bisphosphate (Escobedo et al., 2014). These results provide available evidence for NEDD4L to catalyze ubiquitination of membrane proteins such as SLC7A11. In addition, the mechanism by which NEDD4L regulates SLC7A11 remains to be elucidated, and it is important to determine the motif residues involved in the interaction between SLC7A11 and Ub.

In conclusion, the downregulation of ESR1 leads to a decrease in SLC7A11 expression, resulting in acceleration of IR-induced ferroptosis. Our findings reveal a novel insight that IR-induced ferroptosis was *via* two-way regulation of ESR1/NEDD4L to SLC7A11, and ESR1 might be a critical factor responsible for conferring the resistance to radiotherapy and tumor malignancy. We propose that targeting ESR1 in combination with radiotherapy could enhance the radiotherapeutic efficacy of ER-positive cancers.

## Data Availability

The original contributions presented in the study are included in the article/Supplementary Material. Further inquiries can be directed to the corresponding authors.

## Abbreviations

CI, confidence interval; COX, Cox proportional-hazards model; DEGs, differentially expressed genes; DMSO, dimethyl sulfoxide; ER, estrogen receptor; ESR1, estrogen receptor 1; Fer-1, Ferrostatin-1; GSH, glutathione; HER2, human epidermal growth factor receptor 2; HR, hazard ratio; IP, immunoprecipitation; IR, ionizing radiation; MG132, carbobenzoxy-leu-leu-leucinal; NT, solid tissue normal; OS, overall survival; PR, progesterone receptor; ROS, reactive oxygen species; SD, standard deviation; SLC7A11, solute carrier family 7 member 11; TCGA, the cancer genome atlas; TNBC, triple-negative BRCA; TP, primary solid tumor; qRT-PCR, real-time quantitative reverse transcription PCR; UB, ubiquitin; WCL, whole cell lysate.
