# Supplementary figures and images for: The Dual Regulation Effects of ESR1/NEDD4L on SLC7A11 in Breast Cancer Under Ionizing Radiation
Source: Front Cell Dev Biol. 2022 Feb 16;9:772380. doi: 10.3389/fcell.2021.772380 (PMC8888677; doi:10.3389/fcell.2021.772380)

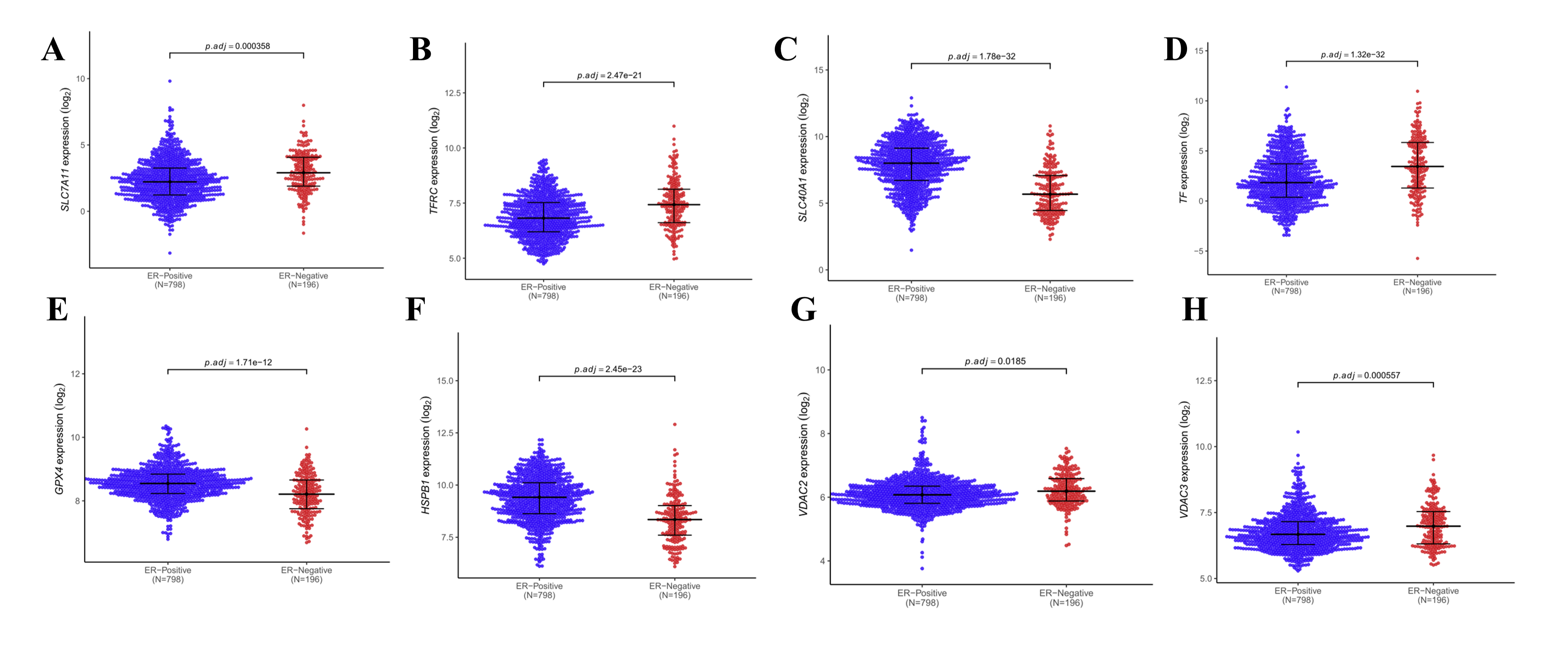

Supplement: Supplementary file 1 [file Image1.tif]
