# Supplementary material for: The Dual Regulation Effects of ESR1/NEDD4L on SLC7A11 in Breast Cancer Under Ionizing Radiation
Source: Front Cell Dev Biol. 2022 Feb 16;9:772380. doi: 10.3389/fcell.2021.772380 (PMC8888677; doi:10.3389/fcell.2021.772380)
Supplement: Supplementary file 3 [file DataSheet1.docx]

Tab. S1 Correlations of SLC7A11 expression in BRCA tissues with clinicopathologic features.

| Clinical characteristics | Variable | SLC7A11 expression | | χ^2^ | *p* |
| --- | --- | --- | --- | --- | --- |
|  |  | High n(%) | Low n(%) |  |  |
| Age | <60 | 162(50.63%) | 363(58.36%) | 5.12 | 0.023 |
|  | >=60 | 158(49.38%) | 259(41.64%) |  |  |
| Molecular subtype | Luminal A | 132(51.16%) | 305(67.18%) | 28.88 | <0.001 |
|  | Luminal B | 44(17.05%) | 79(17.40%) |  |  |
|  | HER2 (+) | 17(6.59%) | 20(4.41%) |  |  |
|  | TNBC | 65(25.19%) | 50(11.01%) |  |  |
| Histological type | Infiltrating Ductal Carcinoma | 255(78.46%) | 417(65.67%) | 34.20 | <0.001 |
|  | Infiltrating Lobular Carcinoma | 32(9.85%) | 164(25.83%) |  |  |
|  | Other | 38(11.69%) | 54(8.50%) |  |  |
| ER | Positive | 237(72.70%) | 554(87.52%) | 32.71 | <0.001 |
|  | Negative | 89(27.30%) | 79(12.48%) |  |  |
| PR | Positive | 197(60.62%) | 487(76.94%) | 28.01 | <0.001 |
|  | Negative | 128(39.39%) | 146(23.07%) |  |  |
| HER2 | Positive | 61(23.64%) | 100(21.98%) | 0.26 | 0.609 |
|  | Negative | 197(76.36%) | 355(78.02%) |  |  |
| T classification | T1 | 75(23.01%) | 169(26.70%) | 3.97 | 0.264 |
|  | T2 | 195(59.82%) | 361(57.03%) |  |  |
|  | T3 | 41(12.58%) | 86(13.59%) |  |  |
|  | T4 | 15(4.60%) | 17(2.69%) |  |  |
| N classification | N0 | 170(52.96%) | 275(44.28%) | 11.28 | 0.010 |
|  | N1 | 93(28.97%) | 227(36.55%) |  |  |
|  | N2 | 42(13.08%) | 67(10.79%) |  |  |
|  | N3 | 16(4.98%) | 52(8.37%) |  |  |
| M classification | M0 | 283(98.61%) | 508(97.13%) | 1.17 | 0.279^*^ |
|  | M1 | 4(1.39%) | 15(2.87%) |  |  |
| Tumor stage | Stage I | 49(15.22%) | 113(18.26%) | 2.73 | 0.434 |
|  | Stage II | 192(59.63%) | 349(56.38%) |  |  |
|  | Stage III | 77(23.91%) | 143(23.10%) |  |  |
|  | Stage IV | 4(1.24%) | 14(2.26%) |  |  |
| Vital status | Alive | 273(85.31%) | 545(87.62%) | 0.99 | 0.321 |
|  | Dead | 47(14.69%) | 77(12.38%) |  |  |

Notes: * Pearson's Chi-squared test with Yates' continuity correction.

Tab. S2 Cox proportional hazards regression model analysis of overall survival

| Variables | Univariate analysis | | Multivariate analysis | |
| --- | --- | --- | --- | --- |
|  | HR (95% CI) | *P* | HR (95% CI) | *P* |
| SLC7A11 (Low vs. High) | 0.69(0.48,0.99) | 0.045 | 0.60(0.41,0.88) | 0.008 |
| age | 1.96(1.42,2.72) | <0.001 | 2.56(1.74,3.78) | <0.001 |
| N classification (N1 vs. N0) | 1.87(1.27,2.77) | 0.002 | 1.90(1.24,2.93) | 0.004 |
| (N2 vs. N0) | 2.72(1.63,4.55) | <0.001 | 3.01(1.72,5.26) | <0.001 |
| (N3 vs. N0) | 4.06(2.25,7.35) | <0.001 | 3.48(1.81,6.69) | <0.001 |
| Histological type (Infiltrating Lobular Carcinoma vs. Infiltrating Ductal Carcinoma) | 0.85(0.54,1.33) | 0.473 |  |  |
| (Other vs. Infiltrating Ductal Carcinoma) | 1.26(0.78,2.01) | 0.342 |  |  |
| ER (Positive vs. Negative) | 0.72(0.50,1.04) | 0.081 |  |  |
| PR (Positive vs. Negative) | 0.76(0.54,1.06) | 0.106 |  |  |
| Molecular subtype (Luminal A vs. HER2 (+)) | 0.46(0.19,1.09) | 0.078 |  |  |
| (Luminal B vs. HER2 (+)) | 0.75 (0.29,1.93) | 0.550 |  |  |
| (TNBC vs. HER2 (+)) | 0.82(0.32,2.06) | 0.671 |  |  |

Tab.S3 E3 ubiquitin ligases targeting SLC7A11 from UbiBrowser.

| E3 | E3GENE | HOMO | PFAM | GO | NET | MOTIF | SCORE | LEVEL |
| --- | --- | --- | --- | --- | --- | --- | --- | --- |
| Q96PU5 | NEDD4L | 1 | 1 | 5.73 | 1 | 6.61 | 0.829 | HIGH |
| Q86TM6 | SYVN1 | 1 | 1 | 1.25 | 1 | 6.61 | 0.714 | MIDDLE |
| Q86YT6 | MIB1 | 1 | 1 | 2.33 | 1 | 2.8 | 0.693 | MIDDLE |
| P46934 | NEDD4 | 1 | 1 | 5.73 | 1 | 1 | 0.681 | MIDDLE |
| P62879 | GNB2 | 1 | 1 | 5.73 | 1 | 1 | 0.681 | MIDDLE |
| P22681 | CBL | 1 | 1 | 3.98 | 1.29 | 1 | 0.671 | MIDDLE |


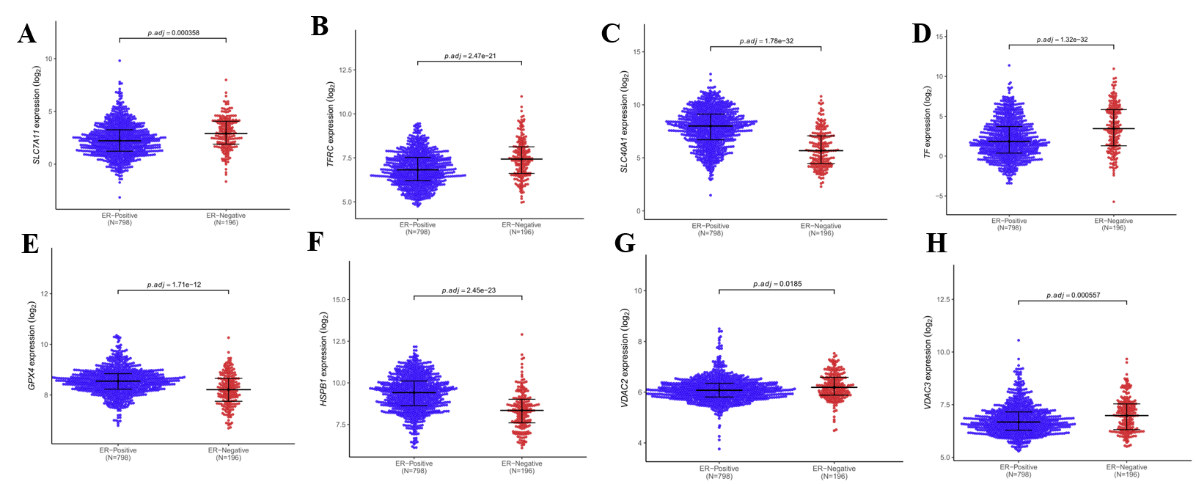


**Fig.S1** Ferroptosis related genes SLC7A11 was downregulated in  ER-positive Compared with ER-negative breast cancer. The verification of the transcriptional expression of vital ferroptosis-related in ER-positive and ER-negative cancers. **(A)** SLC7A11, **(B)** TFRC, **(C)** SLC40A1, **(D)** TF, **(E)** GPX4, **(F)** HSPB1, **(G)** VDAC2, **(H)** VDAC3.
